# Clinical and Economic Outcomes Associated with Cell-Based Quadrivalent Influenza Vaccine vs. Standard-Dose Egg-Based Quadrivalent Influenza Vaccines during the 2018–19 Influenza Season in the United States

**DOI:** 10.3390/vaccines9020080

**Published:** 2021-01-23

**Authors:** Girishanthy Krishnarajah, Victoria Divino, Maarten J. Postma, Stephen I. Pelton, Vamshi Ruthwik Anupindi, Mitch DeKoven, Joaquin Mould-Quevedo

**Affiliations:** 1 Seqirus USA Inc., Summit, NJ 07901, USA; Shanthy.Krishnarajah@seqirus.com; 2 IQVIA, Falls Church, VA 22042, USA; victoria.divino@iqvia.com (V.D.); ruthwik.anupindi@iqvia.com (V.R.A.); Mitch.DeKoven@iqvia.com (M.D.); 3 Unit of PharmacoTherapy, Epidemiology & Economics (PTE2), Department of Pharmacy, University of Groningen, 9700 Groningen, The Netherlands; m.j.postma@rug.nl; 4 Department of Health Sciences, University Medical Centre Groningen (UMCG), University of Groningen, 9700 Groningen, The Netherlands; 5 Department of Economics, Econometrics & Finance, Faculty of Economics & Business, University of Groningen, 9700 Groningen, The Netherlands; 6 Department of Epidemiology, Boston University Schools of Medicine and Public Health, Boston, MA 02118, USA; spelton@bu.edu; 7 Maxwell Finland Laboratories, Boston Medical Center, Boston, MA 02118, USA

**Keywords:** influenza vaccine, cell-based, egg-based, relative vaccine effectiveness, retrospective studies, economic assessment, real-world evidence

## Abstract

Non-egg-based influenza vaccines eliminate the potential for egg-adapted mutations and potentially increase vaccine effectiveness. This retrospective study compared hospitalizations/emergency room (ER) visits and all-cause annualized healthcare costs among subjects aged 4–64 years who received cell-based quadrivalent (QIVc) or standard-dose egg-based quadrivalent (QIVe-SD) influenza vaccine during the 2018–19 influenza season. Administrative claims data (IQVIA PharMetrics^®^ Plus, IQVIA, USA) were utilized to evaluate clinical and economic outcomes. Adjusted relative vaccine effectiveness (rVE) of QIVc vs. QIVe-SD among overall cohort, as well as for three subgroups (age 4–17 years, age 18–64 years, and high-risk) was evaluated using inverse probability of treatment weighting (IPTW) and Poisson regression models. Generalized estimating equation models among the propensity score matched sample were used to estimate annualized all-cause costs. A total of 669,030 recipients of QIVc and 3,062,797 of QIVe-SD were identified after IPTW adjustments. Among the overall cohort, QIVc had higher adjusted rVEs against hospitalizations/ER visits related to influenza, all-cause hospitalizations, and hospitalizations/ER visits associated with any respiratory event compared to QIVe-SD. The adjusted annualized all-cause total costs were higher for QIVe-SD compared to QIVc ((+$461); *p* < 0.05).

## 1. Introduction

Annual influenza exerts a significant clinical and economic burden on the health care system in the United States (U.S.). According to estimates from the Centers for Disease Control and Prevention (CDC), seasonal influenza in the U.S. has caused 9 to 45 million symptomatic illnesses, 4 to 21 million medical visits, 140,000 to 810,000 hospitalizations, and 12,000 to 61,000 deaths annually during the last 10 influenza seasons (2010–2011 to 2019–20) [1,2,3,4]. Vaccination remains the most cost-effective measure to prevent influenza and related untoward outcomes. The constant mutation of the influenza virus requires annual vaccination for optimal protection. The CDC recommends seasonal influenza vaccination for all individuals ≥6 months of age, with few exceptions. Various factors, including the antibody response to vaccines depending on the human immune system, a mismatch between the circulating virus strain and the vaccine virus strain, and egg adaptation associated with egg-based vaccine production, can affect the effectiveness of vaccines in protecting against influenza. Most of the available influenza vaccines in the U.S. are manufactured by growing influenza viruses in eggs. However, egg-based manufacturing methods can select viruses with adaptive mutations acquired for growth [5,6]. These selected egg-based changes can cause a mismatch between the vaccine virus strains and the circulating viruses reducing the specificity of the immune response to the circulating viruses.

For the 2018–19 influenza season, two non-egg-based vaccines were available in the U.S.—Flucelvax^®^ (QIVc, Seqirus USA Inc., Holly Springs, NC, USA) and Flublok^®^ (Sanofi, Fountain Valley, CA, USA). QIVc is a mammalian cell-based quadrivalent influenza vaccine licensed in individuals 4 years and older. For the 2018–19 influenza season, QIVc included purely mammalian cell-based candidate vaccine viruses (CVVs) for influenza A(H3N2) and both influenza B viruses, while influenza A(H1N1) was egg-derived. Flublok is a recombinant hemagglutinin (rHA) quadrivalent vaccine produced in insect cell culture and is approved for people 18 years and older. The standard dose egg-based influenza vaccines available during the 2018–19 influenza season were mostly quadrivalent (QIVe-SD) [7].

Real-world studies conducted during the A(H3N2)-dominated 2017–2018 influenza season showed a trend favoring the QIVc, as compared to the standard dose egg-based vaccines in preventing influenza-related negative outcomes [8,9,10,11,12]. Unlike the 2017–2018 influenza season where A(H3N2) was predominant, the 2018–19 influenza season had A(H1N1)pdm09 viruses predominant from October 2018–mid-February 2019 and A(H3N2) viruses dominated from February 2019 through the end of the season. Due to the seasonal variability of the influenza epidemiology [13,14,15], it is imperative to generate the annual evidence of relative effectiveness (rVE) of available influenza vaccines. Real-world studies provide an opportunity to conduct robust, cost-effective, and timely analyses every season to understand vaccine effectiveness. For the 2018–19 influenza season, there is only one published retrospective study that compared hospitalizations/ER visits associated with cell-based and egg-based influenza vaccines among the U.S. population aged 65 years and above [16]. However, QIVc is licensed for use in the U.S. for individuals 4 years and older and there is limited evidence comparing influenza-related outcomes between QIVc and QIVe-SD among individuals under 65 for the 2018–19 influenza season. To that end, the objective of the current study was to compare the clinical and economic outcomes associated with QIVc vs. QIVe-SD among individuals aged 4–64 years. Flublok was not included in this analysis due to limited vaccinated subjects during the 2018–19 influenza season in our dataset (~2% of all subjects vaccinated with QIVc, QIVe-SD, or Flublok). The study evaluated rVE of QIVc vs. QIVe-SD against influenza-related hospitalizations/ER visits, all-cause hospitalizations, and hospitalizations/ER visits related to any respiratory event during the 2018–19 influenza season in the U.S. The economic outcomes included all-cause annualized costs among QIVc vs QIVe-SD recipients.

## 2. Materials and Methods

### 2.1. Study Overview

This was a retrospective cohort study of QIVc or QIVe-SD vaccine recipients aged 4 to 64 years during the 2018–19 influenza season in the U.S identified using commercial claims database. This study did not require ethics approval due to the retrospective nature utilizing secondary data with de-identified subjects [17,18]. We followed the similar methods utilized in our previously published study for the 2017–18 influenza season [9].

### 2.2. Data Sources

We used the IQVIA PharMetrics^®^ Plus (IQVIA, Denham, NC, USA) data asset. This commercial database consists of claims for 150 million unique enrollees. The database includes information related to demographic profiles of the enrollees, health plan enrollment and payer type, as well as clinical data (e.g., diagnosis/procedures listed on inpatient and outpatient admissions), prescriptions (retail and mail order), and payments. The data are de-identified and Health Insurance Portability and Accountability Act (HIPAA) compliant.

### 2.3. Study Periods

For the purposes of this study, we defined the 2018–2019 influenza season from 1 August 2018 (based on the monthly observed distribution of vaccination) to 31 July 2019. The study period was defined from 1 February 2018 (allowing for a 6-month pre-index period prior to the start of the influenza period) through 31 July 2019. The study selection window to identify individuals who received QIVc or QIVe-SD was from 1 August 2018 through 31 January 2019 [16]. An individual was assigned to a vaccine cohort based on their first claim during the study selection window, and the date of the first claim was termed the ‘index date’. The fixed 6-month pre-index period was used to assess study eligibility criteria, as well as to measure the baseline characteristics of study subjects. The study outcomes assessment period included a variable post-index period that started 14 days after the index date (allowing for the development of vaccine-specific immunity) through the end of the influenza season (31 July 2019).

### 2.4. Study Population

**Figure 1:**
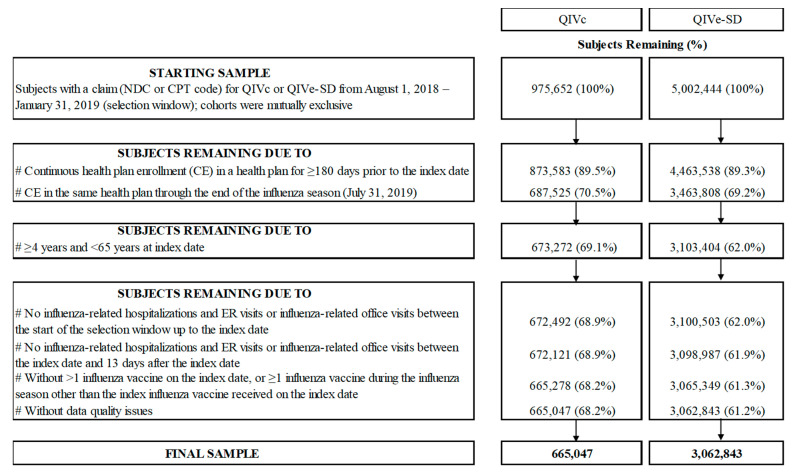
Patient Attrition

Individuals with one or more medical or pharmacy claim that QIVc or QIVe-SD were assigned to two mutually exclusive cohorts (Figure 1). Continuous enrollment ≥180 days prior to the index date up to the end of the study period was required. Furthermore, individuals aged 4 and 64 years at index date were included in the study since QIVc is approved among individuals 4 years and older. Moreover, the database used for this study is considered representative of the national, commercially insured population under 65 and it represents less than 4% of the 65+ year-old population in the U.S.

Individuals with hospitalization/ER visit, or an office visit related to influenza from the start of the influenza season leading up to 13 days post-index date, were excluded. We used the definitions for influenza-related hospitalizations/ER visits (described in more detail in subsequent sections) following similar published methods [8,9,16]. Furthermore, individuals were excluded if they received any other influenza vaccine product or multiple doses (>1 dose) of the index vaccine during the 2018–19 influenza season. Finally, individuals were excluded if they had missing or incomplete data, including invalid/missing birth year, gender, region, health plan enrollment dates, or coverage through Medicare Cost or State Children’s Health Insurance Program (SCHIP).

The subgroup analyses included the following cohorts: (1) individuals aged 4–17 years at index, (2) individuals aged 18–64 years at index, (3) high-risk individuals (those at higher risk for influenza complications). Similar to our previously published study, high-risk individuals were identified based on one or more claim with a diagnosis code, procedure code, or drug code for clinical risk groups, where influenza vaccination is indicated [9]. These clinical risk groups included the asplenia or dysfunction of the spleen, diabetes, chronic kidney disease, chronic heart disease, chronic liver dysfunction, chronic neurological disorders, chronic respiratory disease, immunosuppression, morbid obesity, and pregnancy. The recently published international guidelines, as well as relevant published studies, were utilized to derive these high-risk clinical groups [19,20,21].

### 2.5. Study Measures

The following demographic characteristics were evaluated at the index date—age, gender, geographic region, U.S. Department of Health and Human Services (DHHS) region, type of health plan, and payer type. Clinical characteristics measured over the 6-month pre-index period (excluding the index date, unless otherwise specified) included influenza vaccination month, Charlson Comorbidity Index (CCI Dartmouth–Manitoba adaptation based on ICD-9-CM and ICD-10-CM diagnosis codes), select comorbidities of interest, pre-index HCRU, and pre-index all-cause costs.

#### Study Outcomes

Clinical outcomes included hospitalizations/ER visits related to influenza (ICD-9 487x, 488 x, ICD-10 J09 x, J10 x, J11 x), hospitalizations/ER visits related to respiratory events including pneumonia, asthma/COPD/bronchial events, and any respiratory event (ICD-9-CM 460 x -519 x; ICD-10-CM: J x x), and all-cause hospitalizations.

In addition to the above clinical outcomes, the current study also included hospitalizations/ER visits related to a urinary tract infection (UTI) as a negative control outcome, following the approach of a recently published study [22]. As we do not expect either influenza vaccine to prevent UTI, reporting a negative control outcome can be used to demonstrate similar treatment effects across the two vaccines and control for unmeasured confounding. UTI-related hospitalization/ER visits were defined as a hospitalization or an ER visit with a diagnosis code (ICD-9-CM and ICD-10) in any position [22].

All-cause annualized costs were assessed over the variable follow-up period, starting 14 days after the index date through the end of the influenza season. Total annualized costs included annualized inpatient cost, annualized outpatient medical cost, annualized ER cost, and annualized outpatient pharmacy cost.

### 2.6. Statistical Analysis

#### 2.6.1. Clinical Outcomes Evaluation

Descriptive statistics included means and standard deviations for continuous variables and frequencies for categorical variables. Variables were considered statistically different if standardized mean differences (SMDs) between study cohorts (difference in means or proportions of a variable divided by the pooled standard deviation) were ≥0.10 [23].

Following the prior published work, the statistical approach in the current study followed similar methods [8,9,16]. The inverse probability of treatment weighting (IPTW) was used to adjust for imbalances in measured confounders between vaccine groups [24,25]. Specific details related to the IPTW methods can be found in Divino et al. [9]. Poisson regression models followed by the IPTW adjustments allowed a more robust regression adjustment, thereby reducing any potential bias due to residual confounding. Adjusted rate ratios (RR) and 95% confidence intervals (CIs) for QIVc versus QIVe-SD were estimated using IPTW-weighted multivariate Poisson regression models. Adjusted rVE ([1-RR] * 100%) along with corresponding 95% CIs were calculated for each clinical outcome of interest. Clinically relevant variables with SMD ≥ 0.10 in the pre-IPTW sample that were not included in the IPTW adjustments were also incorporated in the Poisson regression models.

In the subgroup analysis, unadjusted and adjusted clinical outcomes were evaluated for each subgroup of interest: aged 4–17 years, aged 18–64 years, and high-risk cohort. Separate IPTW models were constructed for each subgroup.

#### 2.6.2. Sensitivity Analysis

Sensitivity analysis was conducted by restricting the observation period to the high influenza activity period (HIAP) for the following clinical outcomes—influenza-related hospitalization/ER visits and any respiratory hospitalization/ER visits. The study follow-up period reflecting the HIAP was 23 December 2018—30 March 2019 (Week 52 to 13). An R Language implementation of the moving epidemic method (MEM) algorithm was applied using the R package ‘mem’ to establish epidemic thresholds for the start and end of the influenza season [26]. Epidemic thresholds were calculated using surveillance data from the CDC on the proportion of general physician (GP) visits due to lab-confirmed influenza from 2003/04 through 2017/18 influenza seasons [27]. The proportion of GP visits due to lab-confirmed influenza was above the epidemic thresholds from week 52 through week 13 during the 2018–19 influenza season.

#### 2.6.3. Economics Outcomes Evaluation

The economic outcomes for QIVc versus QIVe-SD were assessed only for the overall cohort (4–64 years). Imbalances in measured confounders between vaccine groups were adjusted using propensity score matching (PSM) methods. The PSM models are widely utilized in observational studies to create more comparable groups [28]. Logistic regression models were used to calculate the propensity score (defined as a probability of receiving QIVc) for each individual. The matching technique included a 1:1 greedy nearest neighbor matching without replacement, using caliper widths of 0.001 of the standard deviation of the logit of the propensity score. The models included all baseline characteristics with absolute SMD ≥ 0.10.

The variable follow-up period that started 14 days after the index date through the end of the influenza season was utilized to assess annualized all-cause costs. Note that since the index date was not included in the economic assessment, costs of index vaccines were excluded from the calculations. All-cause annualized costs were calculated on a per-patient basis and were averaged across the study cohort. We used paired t-test (mean) and the Wilcoxon signed-rank test for continuous variables (median) and McNemar’s test for categorical variables to compare the outcomes across study cohorts. For more robust regression models to further reduce bias due to residual confounding, we used generalized estimating equation models (GEEs) following PSM adjustments. The predicted costs were estimated using recycled prediction techniques [29].

The following predicted annualized all-cause mean costs were estimated: (1) total costs, (2) inpatient costs, (3) outpatient medical costs, (4) ER costs (a subset of outpatient medical costs), and (5) outpatient pharmacy costs. A GEE model with log link function and gamma distribution was developed for total costs and outpatient medical costs. We adjusted for any outliers by limiting the respective cost at the 99th percentile [30]. Due to less frequent outcomes, inpatient costs, ER costs, and outpatient pharmacy costs were estimated using two-part GEE models. In the first part, a GEE model with binomial distribution and logit link was used to estimate the odds of having an outcome (i.e., non-zero costs for the outcome). The second GEE model with gamma distribution and log link estimated the cost of the outcome of interest, among those with the outcome of interest. Outliers were capped at the 99th percentile among patients with at least one such outcome for hospitalizations and ER visits and among all patients for pharmacy. The parameter estimates of GEEs were used to estimate predicted recycled means and 95% CIs were obtained through bootstrapping (500 replications). Finally, any clinically relevant variables that were not included in the PSM because they were well-balanced (SMD ≥ 0.10) in the pre-PSM sample were included in the GEE models.

All analyses for this study were performed using SAS^®^ Software Release 9.3 (SAS Institute Inc., Cary, NC, USA).

## 3. Results

### 3.1. Study Sample

During the 2018–19 influenza season, a total of 5,978,096 vaccine recipients were identified as having at least one claim for either QIVc or QIVe-SD (QIVc = 975,652; QIVe-SD = 5,002,444). After applying the study inclusion and exclusion criteria, the final unadjusted sample included 665,047 QIVc and 3,062,843 QIVe-SD recipients (Figure 1).

### 3.2. Baseline Characteristics

Unadjusted baseline demographic and clinical characteristics for each study cohort are presented in Supplementary Tables S1 and S2. Prior to IPTW, several baseline characteristics were imbalanced with (absolute) SMD ≥ 0.1. For example, QIVc recipients were older than QIVe-SD recipients, with mean (±standard deviation) age of 41.9 (±16.5) and 35.8 (±19.6) years, respectively. The proportion of Medicaid enrollees was lower among QIVc recipients as compared to QIVe-SD recipients (0.1% vs. 0.9%). More QIVc recipients were located in the South (51.6% and 36.2%), while the Midwest region had fewer QIVc recipients as compared to QIVe-SD recipients (23.7% vs. 35.4%). In the 6-month baseline period, QIVc subjects had higher mean outpatient pharmacy costs ($1411 and $1268). After IPTW adjustments, study cohorts were balanced with SMD < 0.10 for all study covariates (n= 669,030 QIVc and 3,062,797 QIVe-SD). Post-IPTW baseline demographic and clinical characteristics are reported in Table 1 and Table 2, respectively.

### 3.3. Clinical Outcomes

**Figure 2:**
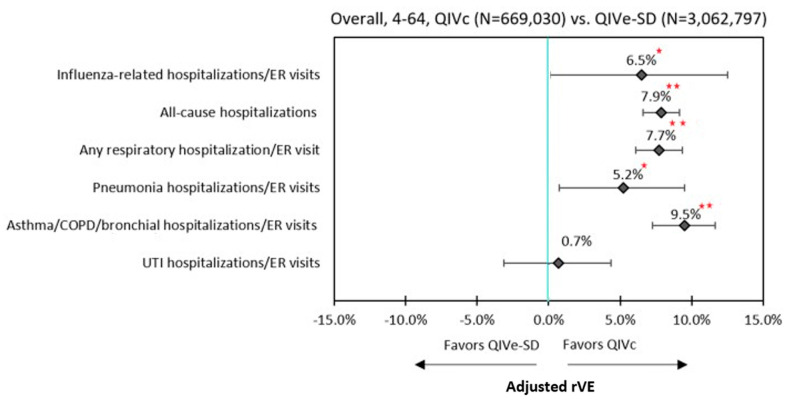
Adjusted rVE –Overall (4–64 Years Old). ** *p* < 0.0001; * *p* < 0.05; ER = emergency room; IPTW = inverse probability of treatment weighting; QIVc = cell-based quadrivalent influenza vaccine; QIVe-SD = standard-dose egg-based quadrivalent influenza vaccine; rVE = relative vaccine effectiveness.

Unadjusted rVEs are presented in Supplementary Table S3. Following IPTW and Poisson regression adjustment, QIVc was more effective than QIVe-SD against influenza-related hospitalizations/ER visits (6.5%; 95% confidence intervals (CI): 0.1–12.5%), all-cause hospitalizations (7.9%, 95% CI: 6.6–9.1%), and hospitalizations/ER visits related to any respiratory event (7.7%; 95% CI: 6.1–9.4%) (Figure 2)

**Figure 3:**
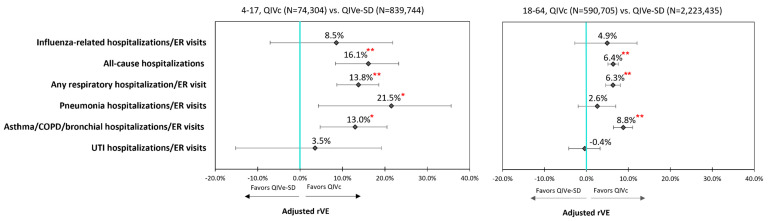
Adjusted rVE: by Age Groups. ** *p* < 0.0001; * *p* < 0.05; ER = emergency room; IPTW = inverse probability of treatment weighting; QIVc = cell-based quadrivalent influenza vaccine; QIVe-SD = standard-dose egg-based quadrivalent influenza vaccine; rVE = relative vaccine effectiveness.

**Figure 4:**
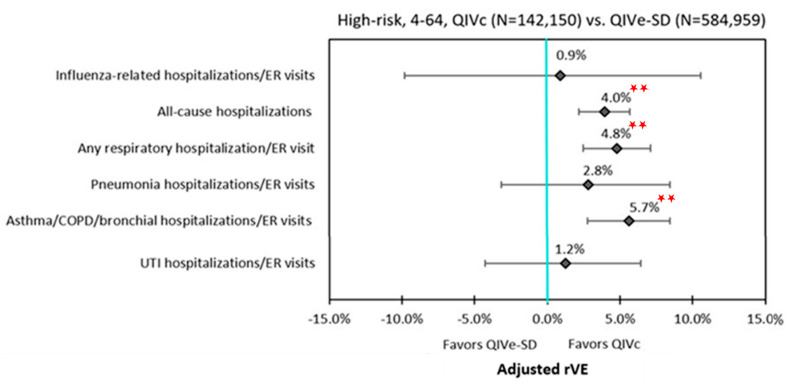
Adjusted rVE: High-risk group. ** *p* < 0.0001; ER = emergency room; IPTW = inverse probability of treatment weighting; QIVc = cell-based quadrivalent influenza vaccine; QIVe-SD = standard-dose egg-based quadrivalent influenza vaccine; rVE = relative vaccine effectiveness.

In the subgroup analysis, QIVc remained more effective than QIVe-SD against all-cause hospitalizations and any respiratory hospitalization/ER visit for age groups 4–17 years, 18–64 years, and the high-risk population. The point estimates for rVEs for influenza-related hospitalizations/ER visits all favored QIVc, but were not statistically significant between QIVc and QIVe-SD among individual subgroups. Similar to the overall cohort, QIVc was significantly more effective than QIVe-SD in preventing hospitalizations/ER visits related to pneumonia (21.5%; 95% CI: 4.3–35.6%) and asthma/COPD/bronchial events (13.0%; 95% CI: 4.7–20.5%) within the 4–17 subgroup. While rVEs for hospitalizations/ER visits related to asthma/COPD/bronchial events remained significantly higher for QIVc than QIVe-SD across all subgroups, a similar effect was not seen for hospitalizations/ER visits related to pneumonia events among 18–64 years age-group and the high-risk cohort (Figure 3 and Figure 4, Table 3).

### 3.4. Sensitivity Analyses

**Figure 5:**
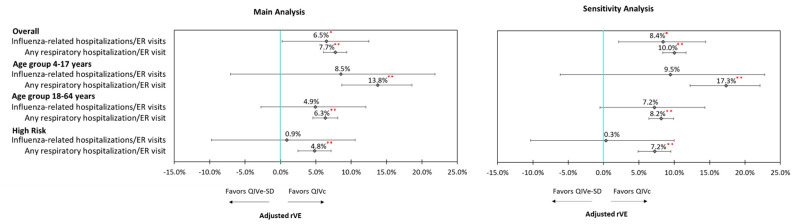
Sensitivity analysis. ** *p* < 0.0001; * *p* < 0.05; ER = emergency room; IPTW = inverse probability of treatment weighting; QIVc = cell-based quadrivalent influenza vaccine; QIVe-SD = standard-dose egg-based quadrivalent influenza vaccine; rVE = relative vaccine effectiveness.

Sensitivity analyses were performed for select clinical outcomes by restricting the observation period to HIAP (23 December 2018–30 March 2019). Results from the sensitivity analyses were consistent with the primary analyses for the overall cohort, as well as for the subgroups. For example, after IPTW adjustment and Poisson regression, QIVc was more effective in reducing influenza-related hospitalizations/ER visits (rVE = 8.4%; 95% CI: 1.4–15.0%) and any respiratory hospitalizations/ER visits (rVE = 10.1%; 95% CI: 7.6–12.4%) compared to QIVe-SD during HIAP. Similar to the main analysis, QIVc was more effective in reducing any respiratory hospitalization/ER visit across both the age groups and high-risk cohort; however, the results were not statistically significant for influenza-related hospitalizations/ER visits (Figure 5).

### 3.5. Economic Outcomes

For the economic analyses, 665,042 QIVc recipients were matched to 665,042 QIVe-SD recipients using PSM approach. All covariates were well-balanced following PSM (Figure 2). Following GEE adjustment, QIVe-SD was associated with significantly higher (+$461) predicted mean per patient annualized all-cause total costs compared to QIVc recipients ($9738 vs. $9277, *p* < 0.0001) (Table 4). Significantly higher outpatient medical costs ($4375 vs. $4612, *p* < 0.001) and inpatient hospitalizations costs ($1559 vs. $1695, *p* < 0.0001) were the primary drivers of higher overall costs for QIVe-SD recipients compared to QIVc recipients.

## 4. Discussion

This real-world study, including over 3.5 million Americans aged 4–64 years who received either QIVc or QIVe-SD vaccine during the 2018–19 influenza season, showed that the relative effectiveness of the QIVc was approximately 7% higher than that of the QIVe-SD in preventing influenza-related hospitalizations/ER visits. Compared to QIVe-SD, QIVc offered significantly higher effectiveness in preventing all-cause hospitalizations, hospitalizations/ER visits related to any respiratory events including pneumonia and asthma/COPD/bronchial events. Similar trends were observed for the study subgroups (aged 4–17 and 18–64 and high-risk) for all-cause hospitalizations/ER visits. We found some variations among the subgroups, particularly for the pneumonia-related hospitalizations/ER visits, where rVE was not significantly different between QIVc and QIVe-SD among 18–64 and high-risk subgroups. Finally, although rVE point estimates were in favor of QIVc, similar to the overall cohort, no statistical significance was achieved when rVE against influenza-related hospitalizations/ER visits was analyzed across different age groups and high-risk population. Results from the sensitivity analysis restricting the observation period to periods of high flu activity were consistent with the main analysis.

This was a robust analysis of QIVc or QIVe-SD vaccine recipients aged 4–64 years identified from a large administrative commercial claims data and utilized a well-established IPTW method to create comparable study groups. To our knowledge, there is only one previous study that compared rVEs of cell-based and egg-based influenza vaccines for the 2018–19 influenza season in the U.S. However, unlike our study that included vaccine recipients aged 4–64 years old, Izurieta et al. included Medicare Fee-for-Service (FFS) population aged 65 years and older [16]. Izurieta et al. reported a positive rVE favoring QIVc compared to QIVe-SD; however, the results were not statistically significant in preventing influenza-related hospitalizations among an older population during the 2018–19 influenza season (rVE 2.5%, 95% CI: −2.4 to 7.3%). In our analysis, we found moderately higher rVE in preventing influenza-related hospitalizations/ER visits for QIVc, as compared to QIVe-SD for the vaccine recipients aged 4–64 years old. The underlying differences in the study population may explain the differences in the results. We did not include the population over the age of 64 years. Hence, our study is not directly comparable to the Izurieta study that included older individuals aged 65 years old and above.

We observed a significant effect modification (separate exposure effects on the outcome due to another variable [31]) by age on any respiratory hospitalization/ER visit and pneumonia hospitalizations/ER visits, as indicated by a statistically significant test for homogeneity in the pre-IPTW sample (Breslow–Day test: *p* < 0.05), supporting the importance of evaluating outcomes stratified by age group. In the current analysis, we found that during the 2018–19 influenza season, QIVc was associated with a significant protective benefit against pneumonia-related hospitalizations/ER visits for the 4–17 age group only. This is generally consistent with the findings from our 2017–18 analysis [9], where we found a similar significant benefit overall and for the 4–17 age group only. Our results suggest a particular benefit of QIVc against pneumonia-related hospitalizations/ER visits for the 4–17 age group specifically.

While QIVc was associated with a significant rVE against influenza-related hospitalizations/ER visits for the overall cohort, the subgroup analyses did not show significant effects for this outcome in QIVc versus QIVe-SD among 4–17 age group, 18–64 age group, and high-risk individuals, separately. During the 2017–18 influenza season, we had found significantly higher rVEs for QIVc vs. QIVe-SD against influenza-related hospitalizations/ER visits overall and for the 18–64 and high-risk subgroups [9]. We believe that these findings are related in part to the event rates being higher in the 2017–18 influenza season, because it was a high severity season. During the 2018–19 influenza season, influenza-related hospitalizations/ER visits were less frequent, and stratification by subgroups further reduced the power to identify a significant effect; therefore, a larger sample is required to be adequately powered to identify a significant effect. Although not significant, trends suggested a benefit with QIVc with positive rVEs. It is also relevant to note that the 2018–19 influenza season was characterized by A(H3N2) vaccine-circulating strain mismatch [1,2,3,4]. Results from the current study, along with the Izurieta study [16], and studies conducted for the prior influenza season [8,9], potentially suggest that QIVc may offer significantly more protective effect particularly among the adult and high-risk populations during a high intensity season, where there is a better match between the circulating virus and the vaccine virus.

Overall, we found that the rVEs were higher among the pediatric population as compared to the adult population. For the 2018–19 influenza season, Chung et al. reported that clinical benefits associated with vaccination were highest among the younger population, with vaccination preventing as much as 43% of all projected A(H1N1)pdm09-associated hospitalizations among children aged 6 months–4 years [4]. It is also important to note that, in general, the rVEs among the high-risk subgroup were lower than the overall cohort; the rVEs may be diluted due to substantially higher event rates in both the vaccine groups among the high-risk cohort (Supplementary Table S4) and rVEs being dependent on the number of individuals with an event in each group. Previous studies have shown significant variability in influenza virus subtypes by influenza seasons and by age groups [4,32]. Moreover, influenza-related mortality rates are higher among the older population and high-risk subgroups [1,2,3,4]. However, we were unable to include death as an outcome in our study due to limitations of the utilized database. More studies focusing on vaccine effectiveness using a spectrum of endpoints, including mortality across different age groups and high-risk individuals, are needed to better understand the impact of vaccines in different populations across various influenza seasons. Although future research to validate the findings of the current study will help in guiding vaccination strategies during future influenza seasons, the confirmation of our study findings obtained in the previous influenza season provides useful insights on choosing the most appropriate type of vaccine.

To our knowledge, there are no prior studies comparing economic outcomes between QIVc and QIVe-SD during the 2018–19 influenza season in the U.S. In the current study, QIVe-SD was associated with substantially higher annualized all-cause total costs (+$461) as compared to QIVc among propensity score matched sample following GEE regression models. These cost differences were primarily due to higher costs associated with greater frequency of healthcare resource utilization, such as emergency room events and inpatient hospitalizations in the QIVe cohort. Several studies evaluating vaccine effectiveness for influenza season 2017–18 reported a favorable trend for QIVc as compared to QIVe-SD [8,9,10,11,12]. The current study reports important findings regarding the relative effectiveness of vaccines and adds to the limited literature available for the 2018–19 influenza season, where only one study has been published that includes vaccine recipients aged 65+ years.

There are several limitations of this study specific to the study design and data source utilized. First, cohort matching methods such as IPTW and PSM used in this study did not necessarily adjust for imbalance in potential unmeasured confounders. Nonetheless, there were no differences between study cohorts in the negative control outcome of UTI hospitalizations/ER visits after IPTW adjustments, suggesting that IPTW created comparable cohorts (Figure 2, Supplementary Table S3). Second, the administrative claims data do not provide clinical details, as they are primarily collected for the purposes of payment. Therefore, there is a potential for miscoding or misclassification. We relied on ICD-9 and ICD-10 codes for lab-confirmed influenza, perhaps introducing outcome misclassification. However, according to the clinical practice guidelines published by the Infectious Diseases Society of America (IDSA), all patients in the hospital setting should be tested for influenza if they have flu-like symptoms [33]. Hence, we expect that the sensitivity of having influenza is high when there is an ICD code at a hospital/ER setting for influenza. Since the endpoints in the current study are hospitalizations/ER visits, there is a high probability of including the correct influenza subject. Third, requiring continuous enrollment may tend to include a relatively unhealthy population; however, this should not affect the study outcomes, as the effect would be similar across the study cohorts. Finally, these findings may not be generalizable to the uninsured, Medicare, or Medicaid populations, as the study sample comprised individuals who were largely commercially or self-insured.

## 5. Conclusions

During the 2018–19 influenza season, after adjusting for confounders, QIVc had higher rVEs against influenza-related hospitalizations/ER visits, hospitalizations/ER visits related to respiratory events, and all-cause hospitalizations compared to QIVe-SD among individuals aged 4–64 years old. QIVe-SD was associated with significantly higher adjusted annualized per patient all-cause total costs, inpatient hospitalization costs, and outpatient medical costs, as compared to QIVc. Further research is needed to validate these findings across multiple seasons to help guide vaccine strategies during future influenza seasons.

## Figures and Tables

**Table 1:** Post- inverse probability of treatment weighting (IPTW) Baseline Demographic Characteristics.

Characteristic	QIVc (N = 669,030)	QIVe-SD (N = 3,062,797)	SMD ^1^
**Mean Age (years)**	36.8	36.9	0.01
SD	19.2	19.2	
Median	39	40	
**Age group (%)**			
4–8 years	10.0%	9.6%	−0.01
9–17 years	15.3%	14.9%	−0.01
18–49 years	40.1%	40.5%	0.01
50–64 years	34.6%	35.0%	0.01
**Female (%)**	55.5%	55.3%	0.00
**Payer type (%)**			
Commercial	60.8%	60.4%	−0.01
Medicaid	0.7%	0.8%	0.00
Medicare Advantage	0.0%	0.0%	0.00
Self-Insured	38.4%	38.8%	0.01
Unknown	0.0%	0.0%	0.00
**Health plan type (n, %)**			
Consumer directed health care	0.1%	0.1%	0.02
HMO	7.7%	8.4%	0.02
POS	5.4%	6.2%	0.03
PPO	85.9%	84.3%	−0.05
Other/Unknown	0.9%	1.0%	0.02
**Geographic region (n, %)**			
Northeast	15.1%	16.6%	0.04
Midwest	35.1%	33.3%	−0.04
South	39.0%	38.8%	0.00
West	10.8%	11.3%	0.02
**DHHS (U.S. Dept. of Health and Human Services) region (%)**			
Region 1: CT, ME, MA, NH, RI, VT	6.1%	6.6%	0.02
Region 2: NJ, NY, PR, VI	2.7%	2.9%	0.02
Region 3: DE, DC, MD, PA, VA, WV	8.7%	9.0%	0.01
Region 4: AL, FL, GA, KY, MS, NC, SC, TN	23.5%	24.0%	0.01
Region 5: IL, IN, MI, MN, OH, WI	26.1%	24.5%	−0.04
Region 6: AR, LA, NM, OK, TX	13.4%	13.2%	0.00
Region 7: IA, KS, MO, NE	7.6%	7.6%	0.00
Region 8: CO, MT, ND, SD, UT, WY	3.0%	2.8%	−0.01
Region 9: AZ, CA, HI, NV, AS, FS, GU, PU	3.0%	3.2%	0.01
Region 10: AK, ID, OR, WA	6.0%	6.1%	0.01

SMD = standardized mean difference; ^1^ SMD (absolute) ≥ 0.10 shows significant difference; IPTW = inverse probability of treatment weighting; POS = point-of-service; HMO = health maintenance organization; PPO = preferred provider organization; QIVc = cell-based quadrivalent influenza vaccine; QIVe-SD = standard-dose egg-based quadrivalent influenza vaccine; SD = standard deviation.

**Table 2:** Post-IPTW Baseline Clinical Characteristics.

Characteristic	QIVc (N = 669,030)	QIVe-SD (N = 3,062,797)	SMD ^1^
**Month of flu vaccine (%)**			
August	1.9%	1.7%	−0.02
September	17.1%	18.1%	0.03
October	47.4%	47.2%	0.00
November	21.3%	20.8%	−0.01
December	7.6%	7.5%	0.00
January	4.7%	4.7%	0.00
**Charlson Comorbidity Index (CCI) Score: (%)**			
0	83.5%	83.3%	−0.01
1	9.7%	9.8%	0.00
2	4.4%	4.4%	0.00
3+	2.5%	2.5%	0.00
**Mean CCI Score**	0.3	0.3	0.01
SD	0.8	0.8	
Median	0	0	
**Pre-index comorbid conditions of interest (%)**			
Asthma	4.1%	4.1%	0.00
Blood disorders	0.1%	0.1%	0.00
Chronic lung disease	1.6%	1.6%	0.00
Diabetes	6.5%	6.7%	0.01
Heart disease	1.5%	1.5%	0.00
Kidney disorders	1.1%	1.0%	−0.01
Liver disorders	1.5%	1.5%	0.00
Neurological or neurodevelopmental conditions	1.5%	1.5%	0.01
Weakened immune system ^2^	2.6%	2.7%	0.01
IBD	0.6%	0.6%	0.00
Composite of the above	16.8%	17.2%	0.01
**Patients with pre-index hospitalization (%)**	2.0%	2.1%	0.01
**Mean Number of pre-index hospitalizations**	0.0	0.0	0.01
SD	0.2	0.2	
Median	0	0	
**Patients with pre-index ER visit (%)**	7.1%	7.7%	0.02
**Mean Number of pre-index ER visits**	0.1	0.1	0.02
SD	0.4	0.5	
Median	0	0	
**Patients with pre-index outpatient physician office visit (%)**	77.4%	76.4%	−0.02
**Mean Number of outpatient physician office visit**	3.8	3.7	−0.01
SD	7.0	6.5	
Median	2	2	
**Mean pre-index outpatient pharmacy costs**	$1259	$1304	0.01
SD	$7566	$8320	
Median	$59	$61	
**Mean pre-index inpatient costs**	$570	$641	0.01
SD	$7575	$8427	
Median	$0	$0	
**Mean pre-index outpatient medical costs**	$1826	$1869	0.01
SD	$7656	$6769	
Median	$442	$450	
**Mean ER costs**	$119	$136	0.02
SD	$851	$972	
Median	$0	$0	
**Mean TOTAL pre-index all-cause costs ^3^**	$4014	$3732	−0.08
SD	$15476	$15391	
Median	$788	$674	

SMD = standardized mean difference; ^1^ SMD (absolute) ≥ 0.10 shows significant difference;^2^ Including: HIV/AIDS; metastatic cancer and acute leukemia; lung or upper digestive or other severe cancer; lymphatic, head, neck, brain, or major cancer; breast, prostate, colorectal, or other cancer; and disorders of immunity; ^3^ TOTAL = outpatient pharmacy + inpatient + outpatient medical; CCI = Charlson comorbidity index score; ER = emergency room; IBD = inflammatory bowel diseases (ulcerative colitis and Crohn’s disease); IPTW = inverse probability of treatment weighting; QIVc = cell-based quadrivalent influenza vaccine; QIVe-SD = standard-dose egg-based quadrivalent influenza vaccine; SD = standard deviation.

**Table 3:** Adjusted rVE—Post-IPTW and Poisson Regression—QIVc vs. QIVe-SD.

Subgroup	Overall (4–64 Years)		4–17 Years		18–64 Years		High-Risk (4–64 Years)	
	rVE	*p*-Value	rVE	*p*-Value	rVE	*p*-Value	rVE	*p*-Value
**Influenza-related hospitalizations and ER visits**	6.5%	0.0475	8.5%	0.2664	4.9%	0.2024	0.9%	0.8611
**All-cause hospitalizations**	7.9%	<0.0001	16.1%	0.0001	6.4%	<0.0001	4.0%	<0.0001
**Respiratory hospitalizations/ER visits**								
Any respiratory hospitalization/ER visit	7.7%	<0.0001	13.8%	<0.0001	6.3%	<0.0001	4.8%	<0.0001
Pneumonia hospitalizations/ER visits	5.2%	0.0234	21.5%	0.0165	2.6%	0.2617	2.8%	0.3435
Asthma/COPD/bronchial hospitalizations/ER visits	9.5%	<0.0001	13.0%	0.0026	8.8%	<0.0001	5.7%	0.0001
**UTI hospitalizations/ER visits**	0.7%	0.7211	3.5%	0.6921	−0.4%	0.8329	1.2%	0.6522

ER = emergency room; IPTW = inverse probability of treatment weighting; QIVc = cell-based quadrivalent influenza vaccine; QIVe-SD = standard-dose egg-based quadrivalent influenza vaccine; rVE = relative vaccine effectiveness.

**Table 4:** Economic Outcomes—Post-PSM and GEE Adjustment.

Predicted Mean Annualized All-Cause Cost	QIVc N = 665,042		QIVe-SD N = 665,042		*p*-Value	Incremental Mean
	Mean	95% Cis *	Mean	95% Cis *		
TOTAL	$9277	$9168–$9391	$9738	$9620–$9854	<0.0001	$461
Inpatient	$1559	$1532–$1589	$1695	$1667–$1725	<0.0001	$136
Outpatient medical	$4375	$4348–$4404	$4612	$4583–$4644	<0.0001	$238
ER	$261	$258–$265	$291	$288–$295	<0.0001	$30
Outpatient pharmacy	$3182	$3068–$3305	$3193	$3079–$3317	0.260	$11

CIs = confidence intervals; ER = emergency room; GEE = generalized estimating equation; PSM = propensity score matching; QIVc = cell-based quadrivalent influenza vaccine; QIVe-SD = standard-dose egg-based quadrivalent influenza vaccine. * Non-overlapping confidence intervals indicate statistical significance.

## Data Availability

The original de-identified data used in this analysis were obtained from and are the property of IQVIA. IQVIA has restrictions prohibiting the authors from making the data set publicly available. Interested researchers may contact IQVIA to apply to gain access to the study’s data in the same way the authors obtained the data (see https://www.iqvia.com/contact/sf).

## Supplementary Materials

The following are available online at https://www.mdpi.com/2076-393X/9/2/80/s1, Table S1: Baseline Demographic Characteristics—Unadjusted, Table S2: Baseline Clinical Characteristics—Unadjusted, Table S3: Unadjusted rVEs, Table S4: Adjusted Clinical Outcomes Rates (Rates per 1000).
