# Supplementary material for: Clinical and Economic Outcomes Associated with Cell-Based Quadrivalent Influenza Vaccine vs. Standard-Dose Egg-Based Quadrivalent Influenza Vaccines during the 2018–19 Influenza Season in the United States
Source: Vaccines (Basel). 2021 Jan 23;9(2):80. doi: 10.3390/vaccines9020080 (PMC7911861; doi:10.3390/vaccines9020080)
Supplement: Supplementary file 1 [file vaccines-09-00080-s001.pdf]

Supplementary Table 1. Baseline Demographic Characteristics – Unadjusted

Characteristic	QIVc (N=665,047)	QIVe-SD (N=3,062,843)	SMD[1]
**Mean Age (years)**	41.9	35.8	-0.31
SD	16.5	19.6	
Median	45	38	
**Age group (%)**			
4-8 years	3.0%	11.0%	0.32
9-17 years	8.1%	16.4%	0.25
18-49 years	47.4%	39.0%	-0.17
50-64 years	41.4%	33.6%	-0.16
**Gender (n,%)**			
Male	43.4%	44.9%	0.03
Female	56.6%	55.1%	-0.03
**Payer type (%)**			
Commercial	62.7%	59.9%	-0.06
Medicaid	0.1%	0.9%	0.11
Medicare Advantage	0.0%	0.0%	0.02
Self-Insured	37.2%	39.2%	0.04
**Health plan type (%)**			
Consumer directed health care	0.0%	0.1%	0.03
HMO	7.6%	8.4%	0.03
POS	4.8%	6.3%	0.07
PPO	87.0%	84.1%	-0.09
Other/Unknown	0.5%	1.1%	0.06
**Geographic region (%)**			
Northeast	14.4%	17.0%	0.07
Midwest	23.7%	35.4%	0.26
South	51.6%	36.2%	-0.32
West	10.3%	11.5%	0.04
**DHHS (U.S. Dept. of Health & Human Services) region (%)**			
Region 1: CT, ME, MA, NH, RI, VT	6.7%	6.6%	-0.01
Region 2: NJ, NY, PR, VI	2.6%	3.0%	0.02
Region 3: DE, DC, MD, PA, VA, WV	7.0%	9.5%	0.09
Region 4: AL, FL, GA, KY, MS, NC, SC, TN	35.6%	21.6%	-0.31
Region 5: IL, IN, MI, MN, OH, WI	16.4%	26.2%	0.24
Region 6: AR, LA, NM, OK, TX	14.4%	13.0%	-0.04
Region 7: IA, KS, MO, NE	6.7%	7.8%	0.04
Region 8: CO, MT, ND, SD, UT, WY	1.3%	3.2%	0.13
Region 9: AZ, CA, HI, NV, AS, FS, GU, PU	2.7%	3.3%	0.03
Region 10: AK, ID, OR, WA	6.6%	6.0%	-0.02

[1] *SMD (absolute) ≥0.1 indicates significance ; HMO = health maintenance organization; POS = point-of-service, PPO = preferred provider organization; QIVc = cell-based quadrivalent influenza vaccine; QIVe-SD = standard-dose egg-based quadrivalent influenza vaccine; SD = standard deviation; SMD = standardized mean difference*

Supplementary Table 2. Baseline Clinical Characteristics – Unadjusted

Characteristic	QIVc (N=665,047)	QIVe-SD (N=3,062,843)	SMD[1]
**Month of flu vaccine (%)**			
August	0.2%	2.0%	0.18
September	13.8%	19.1%	0.14
October	50.2%	46.6%	-0.07
November	20.2%	20.9%	0.02
December	9.5%	7.1%	-0.09
January	6.0%	4.4%	-0.08
**Charlson Comorbidity Index (CCI) Score: (%)**			
0	81.3%	83.7%	0.06
1	10.5%	9.6%	-0.03
2	5.2%	4.3%	-0.05
3+	2.9%	2.4%	-0.04
**Mean CCI**	0.3	0.3	-0.07
SD	0.9	0.8	
Median	0	0	
**Pre-index comorbid conditions of interest (%)**			
Asthma	3.9%	4.2%	0.01
Blood disorders	0.1%	0.1%	0.00
Chronic lung disease	1.9%	1.6%	-0.02
Diabetes	7.8%	6.4%	-0.05
Heart disease	1.8%	1.5%	-0.03
Kidney disorders	1.3%	0.9%	-0.03
Liver disorders	1.8%	1.5%	-0.03
Neurological or neurodevelopmental conditions	1.6%	1.5%	-0.01
Weakened immune system[2]	3.0%	2.6%	-0.02
IBD	0.7%	0.6%	-0.01
Composite of the above	18.8%	16.7%	-0.05
**Patients with pre-index hospitalization (%)**	2.3%	2.1%	-0.01
**Number of pre-index hospitalizations**			
Mean	0.0	0.0	-0.01
SD	0.2	0.2	
Median	0	0	
**Patients with pre-index ER visit (%)**	7.4%	7.7%	0.01
**Mean number of pre-index ER visits**	0.1	0.1	0.01
SD	0.5	0.5	
Median	0	0	
**Patients with pre-index outpatient physician office visit (%)**	78.0%	76.3%	-0.04
**Mean number of pre-index outpatient physician office visits**	3.9	3.7	-0.06
SD	6.8	6.6	
Median	2	2	
**Mean total pre-index all-cause outpatient pharmacy costs**	$1,411	$1,268	-0.13
SD	$7,666	$8,254	
Median	$87	$56	
**Total pre-index all-cause medical costs**			
**Mean inpatient**	$640	$624	-0.01

SD	$7,735	$8,349	
Median	$0	$0	
**Mean outpatient**	$1,963	$1,840	-0.05
SD	$7,811	$6,727	
Median	$485	$441	
**Mean ER**	$133	$132	0.01
SD	$931	$947	
Median	$0	$0	
**Mean TOTAL pre-index all-cause costs[3]**	$4,014	$3,732	-0.08
SD	$15,476	$15,391	
Median	$788	$674	

[1] *SMD (absolute) ≥0.1 indicates significance*

[2] *Including: HIV/AIDS; metastatic cancer and acute leukemia; lung or upper digestive or other severe cancer; lymphatic, head, neck, brain, or major cancer; breast, prostate, colorectal, or other cancer; and disorders of immunity*

[3] *TOTAL = outpatient pharmacy + inpatient + outpatient medical*

CCI = Charlson comorbidity index score; ER = emergency room; IBD = inflammatory bowel diseases (ulcerative colitis and Crohn's disease); IPTW = inverse probability of treatment weighting; QIVc = cell-based quadrivalent influenza vaccine; QIVe-SD = standard-dose egg-based quadrivalent influenza vaccine; SD = standard deviation; SMD = standardized mean difference

Supplementary Table 3. Unadjusted rVEs

Subgroup	**Overall (4-64 years)**		**4-17 years**		**18-64 years**		**High-risk (4-64 years)**	
	**rVE**	**p-value**	**rVE**	**p-value**	**rVE**	**p-value**	**rVE**	**p-value**
**All-cause hospitalizations**	-6.7%	<.0001	14.5%	0.0004	8.9%	<.0001	1.6%	0.0726
**Influenza-related hospitalizations and ER visits**	-14.3%	<.0001	6.3%	0.4718	-0.9%	0.6475	-4.7%	0.0867
**Respiratory hospitalization / ER events**								
Any respiratory hospitalization/ER visit	6.3%	<.0001	22.2%	<.0001	8.1%	<.0001	7.7%	<.0001
Pneumonia hospitalizations/ER visits	-4.5%	0.0553	32.9%	0.0002	3.6%	0.1252	-0.1%	0.9624
Asthma/COPD/bronchial hospitalizations/ER visits	3.4%	0.0043	20.2%	<.0001	10.1%	<.0001	9.0%	<.0001
**Influenza-related hospitalizations and ER visits - SA**	19.4%	<.0001	21.4%	0.0049	7.8%	0.0430	14.1%	0.0054
**UTI hospitalizations/ER visits**	6.9%	<.0001	22.4%	<.0001	7.0%	<.0001	6.1%	<.0001

**SA = Sensitivity Analysis*

Supplementary Table 4. Adjusted Clinical Outcomes Rates (Rates per 1,000)

Age group	Overall		4-17 Years		18-64 years		High-risk	
Study Cohort	QIVc	QIVe-SD	QIVc	QIVe-SD	QIVc	QIVe-SD	QIVc	QIVe-SD
	(N=669,030)	(N=3,062,797)	(N=74,304)	(N=839,744)	(N=590,705)	(N=2,223,435)	(N=142,150)	(N=584,959)
**Measure (rate)**								
**All-cause hospitalizations**	35.6	38.7	7.1	8.6	45.3	48.5	100.0	104.1
**Influenza-related hospitalizations/ER visits**	1.6	1.7	2.3	2.5	1.3	1.4	3.2	3.2
**Respiratory hospitalization/ER events**								
Any respiratory hospitalization/ER visit	21.8	23.7	16.9	19.8	23.2	25.0	56.0	59.0
Pneumonia hospitalizations/ER visits	3.2	3.4	1.4	1.8	3.8	4.0	9.4	9.7
Asthma/COPD/bronchial hospitalizations/ER visits	11.5	12.8	6.8	8.0	13.0	14.3	36.9	39.2
**UTI hospitalizations/ER visits**	4.9	5.0	1.8	2.0	6.0	6.0	11.5	11.6

Rate = events (first event only) per 1,000 vaccinated-subject seasons
